# Utilization of deworming medication and its associated factors among pregnant married women in 26 sub-Saharan African countries: a multi-country analysis

**DOI:** 10.1186/s41182-021-00343-x

**Published:** 2021-06-30

**Authors:** Betregiorgis Zegeye, Mpho Keetile, Bright Opoku Ahinkorah, Edward Kwabena Ameyaw, Abdul-Aziz Seidu, Sanni Yaya

**Affiliations:** 1 HaSET Maternal and Child Health Research Program, Shewarobit Field Office, Shewarobit, Ethiopia; 2 Department of Population Studies, Faculty of Social Sciences, University of Botswana, Private Bag, UB 0022 Gaborone, Botswana; 3 School of Public Health, Faculty of Health, University of Technology Sydney, Sydney, NSW 2007 Australia; 4 Department of Population and Health, University of Cape Coast, Cape Coast, PMB 0494 Ghana; 5 College of Public Health, Medical and Veterinary Sciences, James Cook University, Townsville, QLD 4811 Australia; 6 School of International Development and Global Studies, Faculty of Social Sciences, University of Ottawa, 120 University Private, Ottawa, ON K1N 6N5 Canada; 7 The George Institute for Global Health, Imperial College London, London, UK

**Keywords:** Deworming, DHS, Factors, Global health, Pregnant women, Sub-Saharan Africa, Utilization

## Abstract

**Abstract:**

**Background:**

Deworming is one of the strategies to reduce the burden of anemia among pregnant women. Globally, pregnant women in sub-Saharan Africa are more affected by anemia. Therefore, this study examined both the coverage and demographic, socioeconomic, and women empowerment-related factors associated with the utilization of deworming medication among pregnant married women in sub-Saharan Africa.

**Methods:**

We used data from the most recent Demographic and Health Surveys of 26 countries in sub-Saharan Africa conducted between 2010 and 2019. Using Stata version-14 software, analysis was done on 168,910 pregnant married women. Bivariate and multivariable logistic regression analyses were conducted to examine the factors associated with the utilization of deworming medication. The results were presented using adjusted odds ratios (aORs) at 95% confidence intervals (CIs).

**Results:**

The pooled results showed that about 50.7% (95% CI 48.2–53.3%) of pregnant married women in the studied countries took deworming medications, and this varied from as high as 84.1% in Sierra Leone to as low as 2% in Angola. Regarding sub-regional coverage, the highest and lowest coverages were seen in East Africa (67.6%, 95% CI 66.0–69.1%) and West Africa (24.3%, 95% CI 22.4–26.4%) respectively. We found higher odds of utilization of deworming medication among older pregnant married women (aOR=1.93, 95% CI 1.32–2.84), women with educated husbands (aOR=1.40, 95% CI 1.11–1.77), wealthier women (aOR=3.12, 95% CI 1.95–4.99), women exposed to media (aOR=1.46, 95% CI 1.18–1.80), and those who had four or more antenatal care visits (aOR=1.51, 95% CI 1.24–1.83).

**Conclusions:**

Enhancing women’s education, disseminating information about maternal healthcare services through mass media, and ensuring that women from economically disadvantaged households benefit from national economic growth can be considered as deworming medication improvement strategies in sub-Saharan Africa. Moreover, providing more attention to adolescents or young pregnant women and increasing the number of antenatal care visits could be considered to increase deworming uptake among pregnant married women.

## Background

Soil-transmitted helminths (STH) is one of the major public health problems worldwide [1–6]. Globally, approximately 1.5 billion people are affected by STH [6]. Majority of women of reproductive age (WRA) including pregnant women are the most affected [3, 4, 6]. Most of the disease burden occurs in the tropical and sub-tropical areas of sub-Saharan Africa (SSA) and South East Asia [5]. WRA in South East Asian and African regions are responsible for 74.7% of all STH risk among WRA [3, 4]. Hookworm and whipworm (*Trichuris trichiura*) are the most common helminths among pregnant women [2, 4, 6]. Pregnant women especially are at particular risk for STH [6, 7]. Pregnant women with intestinal parasitic infection are at increased risk of maternal complications and adverse perinatal outcomes such as anemia, low birth weight, and perinatal mortality [8, 9]. Anemia affects approximately 39% of WRA, including pregnant women in SSA [10], which is higher than the average in low- and middle-income countries (LMICs) (35.4%) and worldwide (33%). The prevalence of anemia among pregnant women in SSA is 46% [11].

The World Health Organization (WHO) recommended deworming or preventive chemotherapy for pregnant women after the first trimester, using a single dose of mebendazole (500 mg) or albendazole (400 mg) in areas where there is 40% or higher anemia prevalence among pregnant women and the prevalence of *T. Trichiura* and hookworm is 20% and above [6, 12, 13]. As documented in several prior studies, prevention chemotherapy or deworming of pregnant women and children significantly enhances hemoglobin level and nutritional status [14–16]. Women who are dewormed during pregnancy tend to have an improved birth weight [17, 18] and a reduction in prevalence of low birth weight [19].

In 2001, representatives at the World Health Assembly universally recognized a determination (WHA54.19) urging endemic countries to start seriously tackling worms, specifically schistosomiasis and as a strategy to control STH in high endemic regions such as SSA [6], and WHO prepared PC guideline to control STH especially among 103 STH-endemic countries, and as this is often a worldwide rule, Part States are anticipated to adjust the proposal concurring to their setting and its achievability [20]. In line with this, integrated STH control strategies were designed in many countries in SSA, although the performance is not satisfactory related to limited funds to cascade the program [21].

For instance, a few funds have been committed by the Bill & Melinda Gates Foundation (for backing), the USAID (up to $100 million, with the probability of more in a long time to come), the Geneva Worldwide ($8.9 million), and the British Government (up to £50 million promised for a few NTDs) for ten sub-Saharan African nations, namely Burkina Faso, Burundi, Ghana, Mali, Niger, Rwanda, Sierra Leone, Southern Sudan, Tanzania, and Uganda, to execute their national programs utilizing coordinates of neglected tropical diseases control. In any case, the funds accessible are not however adequate for these nations to scale up totally [21, 22].

To successfully control STH in STH-endemic countries, establishing an efficient STH control program in adolescent, pregnant, and lactating WRA is one of the six targets to be achieved by 2030 [23, 24]. STH control programs have been successful during the 2010–2020 decade, and a number of STH-endemic countries including most SSA countries conducted PC programs for more than 5 years, although few countries such as Botswana have not started the program [24].

As of 2017, the coverage of preventive chemotherapy was <75% in Angola, Chad, Comoros, Democratic Republic of the Congo, Ethiopia, Gabon, and Kenya, whereas Congo, Côte d’Ivoire, Guinea, Liberia, Nigeria, Uganda, Zambia, and Zimbabwe implemented for less than 5 years and achieved ≥ 75%. Similarly, Burundi, Cameroon, Ghana, Malawi, Rwanda, Sierra Leone, and Togo implemented for 5 years and more and achieved ≥ 75% preventive chemotherapy coverage. Few countries in SSA such as Benin, Burkina Faso, Mali, Niger, and Senegal recorded the moderate and heavy intensity less than 1% [24].

Specifically to pregnant women, WHO has set a target to achieve the coverage of deworming at 75% in 2030 [6, 24]. Available evidence shows that majority of pregnant women do not receive deworming medication [5]. For instance, a study in 49 STH endemic countries showed that about 688 million WRA living in STH endemic countries were in need of deworming medication as of 2015. However, about only 23% (95% CI 19–28%) of pregnant women received deworming medication during antenatal care (ANC) [5]. Another recent study in Cameroon shows only 29.8% of pregnant women received deworming medication during pregnancy [25].

Few studies on deworming medication are available in SSA. However, most of the available studies have focused on the estimation of coverage [5, 26], efficacy, safety, and tolerability of deworming medication [17]; effect of mass deworming [7] on schistosomiasis and all age of population [27], deceptive report [28], and on single health facility of a single country [26]. As a result, there is scarcity of evidence about both coverage and utilization of deworming medication and its associated factors among pregnant women in SSA. This study aimed at examining both the coverage and demographic, socio-economic, and women empowerment-related factors associated with the utilization of deworming medication among pregnant married women in 25 countries in SSA. The current study can help policy makers to evaluate the implementing policies and programs or design new policies regarding deworming medication utilization in the region.

## Methods

### Study design

This study was a cross-sectional study, and involved the extraction of data from the Demographic and Health Survey (DHS) of 26 countries in SSA conducted between 2010 and 2019. DHS is a nationally representative survey aimed to collect data on demographic and several health indicators including utilization of deworming medication across many LMICs [29]. DHS was carried out with financial aid from the United States Aid for International Development (USAID) and technical support of Inner-City Fund international [30]. During the DHS, a two-stage stratified cluster sampling technique was applied [31]. In the first stage, enumeration areas (EAs) also called clusters were selected from sampling frame prepared from the recent population census. In the second stage, households (usually 25–30 households) were selected from the clusters. We included 26 countries in SSA because of the inclusion criteria: availability of dependent variable and key explanatory variables and DHS conducted between 2010 and 2019. The analysis was done on 168,910 pregnant married women. The DHS datasets are freely available for download at https://dhsprogram.com/data/available-datasets.cfm. The reason we limited the analysis to married women was the inclusion of women empowerment variables which is applicable to married women as explanatory variables (women’s decision-making power and wife beating attitude) [32, 33]. We followed the guidelines for Strengthening of Observational Studies in Epidemiology (STROBE) while preparing this manuscript [34]. Please refer to Table 1, for more details about studied countries and samples for the study.

**Table 1:** Year of survey for each studied countries and sampled population

Country	Year of survey	Sampled population		Coverage of deworming medication
		Weighted number	Weighted percent	
Angola	2015/16	6361	3.77	2.03
Burkina Faso	2010	9964	5.90	24.31
Benin	2017/18	8331	4.93	64.39
Burundi	2016/17	7621	4.51	67.60
Congo Democratic Republic	2013/14	9551	5.65	55.64
Côte d’Ivoire	2011/12	4495	2.66	37.50
Cameroon	2018/19	5013	2.97	29.82
Ethiopia	2016	6662	3.94	5.73
Gabon	2012	2875	1.70	71.58
Ghana	2014	3631	2.15	39.71
Gambia	2013	4974	2.94	40.82
Guinea	2018	5138	3.04	38.07
Kenya	2014	5904	3.50	31.81
Comoros	2012	1878	1.11	63.16
Liberia	2013	4035	2.39	58.56
Mali	2018	6066	3.59	50.15
Malawi	2016/17	11,070	6.55	51.88
Nigeria	2018	14,185	8.40	51.61
Rwanda	2014/15	4741	2.81	49.98
Sierra-Leone	2019	6090	3.61	84.10
Senegal	2010/11	7571	4.48	25.01
Chad	2014/15	10,248	6.07	23.31
Togo	2013/14	4556	2.70	57.16
Uganda	2016	8429	5.00	60.50
Zambia	2018/19	5496	3.25	77.76
Zimbabwe	2015	4025	2.38	3.57
Total		168,910	100.00	

### Study variables

#### Dependent variable

The dependent variable of this study was utilization of deworming medication. The WHO recommends that a pregnant woman should take a single dose of mebendazole (500 mg) or albendazole (400 mg) after the first trimester [12, 25]. In DHS, women of reproductive age with birth history in the last 5 years preceding the survey are asked whether or not they took deworming medication, and the women responded as yes or no [13, 33]. Then, the dependent variable coded into binary as “yes” if the women took deworming medication and “no” if they did not take the deworming medication.

#### Explanatory variables

Based on available evidence on factors associated with uptake of deworming medication [5, 7, 17–19, 25–27], we included the following explanatory variables and categorized them primarily based on the DHS guide [26] and also used available studies on same topic. These included women’s age (15–19, 20–24, 25–29, 30–34, 35–39, 40–44, 45–49), women’s educational status (no formal education, primary school, secondary school, higher), husband’s educational status (no formal education, primary school, secondary school, higher), women’s occupation (not working, professional/technical/managerial, agricultural, manual, others), family size (<5, 5+), place of residence (urban, rural), religion (Christian, Muslim, others), timing of antenatal care (ANC) visits (≤3 months, >3 months), number of ANC visit (<4 months, ≥4 months), and wanted last birth (wanted then, wanted later, wanted no more). Other variables were wealth index, media exposure, decision-making power and attitude towards wife beating. In DHS, wealth index was computed using durable goods, household characteristics, and basic services following the methodology explained elsewhere [35], and was further categorized into five categories: poorest, poorer, middle, richer, and richest. Media exposure was coded as yes versus no. If the women read newspaper or listened to the radio or watched television for at least less than once a week, we coded as *yes* and coded as *no* otherwise.

In the DHS, married women aged 15–49 are asked three questions to measure their decision-making power within households: who decides about your own health? Who decides about large household purchases? Who decides on visit of families or relatives? The questions are intended to measure the women’s decision-making power so as to indirectly examine whether or not the women are empowered or not. If the woman decided (either alone or together with her husband) on all the three aforementioned decision-making parameters, the woman was considered to have decision-making power, and was coded as 1, and otherwise considered as no decision-making power and coded as 0 [32, 33]. Similarly, in the DHS, women of reproductive age are asked five questions to measure wife beating attitude: do you agree with wife beating when a woman burns food? Do you agree with wife beating when a woman goes out without telling her husband? Do you agree with wife beating when a woman neglects children? Do you agree with wife beating when a woman argues with her husband? Do you agree with wife beating when a woman refuses to have sex with her husband? Then, if a woman responded that a husband was justified for wife beating for at least one of the five reasons, she was considered as justifying wife beating and we coded as 0, and if the woman did not justify/disagreed for all of the five abovementioned wife beating reasons, she was considered as not justifying wife beating and was coded as 1 [32, 33, 36].

### Statistical analyses

Using the Stata version 14.0 software, descriptive analysis such as frequency distribution was carried out to show the prevalence of deworming medication utilization. This was followed by Pearson chi-square test (χ^2^) and multicollinearity test (using variance inflation factor (VIF), mean VIF=1.84, max VIF=4.23, min VIF=1.06). Then, bivariate and multivariable logistic regression analysis followed by testing model fitness (using Hosmer-Lemeshow, P=0.3530) was carried out. P-value less than 0.05 was used as a cutoff point for both bivariate and multivariable logistic regression. To take care of the complex nature of the DHS’s data, we used the “svyset” command during analysis, and all three design elements such as weight, cluster, and strata were taken into consideration.

### Ethical considerations

We used publicly available secondary data for analysis of this study (available at https://dhsprogram.com/data/available-datasets.cfm.). Ethical procedures were done by institutions that fund, commissioned, and managed the surveys, and no further ethical clearance was required. ICF international approved that all the DHS surveys follow the US Department of Health and Human Services rules, for respecting of human subject’s rights. For more details related to ethical issues, readers can visit http://goo.gl/ny8T6X.

## Results

### Sociodemographic characteristics of respondents

As shown in Table 2, a total of 168,910 participants were involved for analysis of this study. Of them, 7.9% of respondents were within 15–19 years while about 27.5% of respondents and 21.1% of their husbands did not attend formal education respectively. Approximately three quarters (25.3%) of the respondents were not working, and 35.3% were rural residents. Meanwhile, 34.6% of married women had not decided for at least one reason of decision-making parameters: their own health, purchase large household expenses, and to visit families or relatives. About 28.6% of married women agreed or justified for wife beating for at least one of the five wife beating reasons.

**Table 2:** Frequency distribution of study participants and deworming distribution across explanatory variables: evidence from 26 sub-Saharan African countries DHSs

Variables	Number (weighted %)	Deworming medication (weighted %)		Chi-square, P-value
		No	Yes	
**Age in years**				χ^2^=75.01, p<0.001
15–19	17,193 (7.86)	62.99	37.01	
20–24	44,508 (19.88)	52.28	47.72	
25–29	55,243 (21.60)	44.80	55.20	
30–34	47,557 (16.88)	46.00	54.00	
35–39	40,591 (14.56)	47.03	52.97	
40–44	29,250 (11.73)	47.94	52.06	
45–49	22,617 (7.51)	63.62	36.38	
**Women’s educational status**				χ^2^=615.81, p<0.001
No formal education	112,216 (27.46)	69.86	30.14	
Primary school	80,241 (38.90)	50.82	49.18	
Secondary school	54,423 (29.65)	30.77	69.23	
Higher	10,069 (3.98)	22.70	77.30	
**Husband’s educational status**				χ^2^=571.32, p<0.001
No formal education	96,580 (21.13)	66.22	33.78	
Primary school	62,425 (26.62)	62.78	37.22	
Secondary school	67,653 (44.91)	36.57	63.43	
Higher	19,207 (7.34)	24.54	75.46	
**Women’s occupation**				χ^2^=662.67, p<0.001
Not working	58,510 (25.30)	42.32	57.68	
Professional/technical/managerial	9,517 (5.41)	33.64	66.36	
Agricultural	79,991 (29.67)	73.21	26.79	
Manual	16,683 (3.39)	40.35	59.65	
Others	74,493 (36.23)	36.48	63.52	
**Wealth index**				χ^2^=1187.29, p<0.001
Poorest	58,177 (17.92)	80.63	19.37	
Poorer	53,642 (20.66)	67.28	32.72	
Middle	50,997 (20.72)	44.33	55.67	
Richer	48,109 (20.59)	29.96	70.04	
Richest	46,034 (20.11)	21.83	78.17	
**Media exposure**				χ^2^=703.03, p<0.001
No	115,882 (28.22)	74.96	25.04	
Yes	140,589 (71.78)	38.51	61.49	
**Family size**				χ^2^= 20.43, p<0.01
<5	78,802 (33.09)	53.6	46.40	
5+	178,157 (66.91)	47.41	52.59	
**Religion**				χ^2^=40.13, p<0.001
Christian	145,994 (93.53)	48.23	51.77	
Muslim	100,493 (0.36)	46.09	53.91	
Others	10,885 (6.11)	64.36	35.64	
**ANC follow-up**				χ^2^=1257.17, p<0.001
No	21,703 (18.33)	96.17	3.83	
Yes	159,944 (81.67)	38.73	61.27	
**Timing of first ANC**				χ^2^=60.41, p<0.001
>3 months	98,455 (51.38)	43.93	56.07	
≤3 months	61,501 (48.62)	33.23	66.77	
**Number of ANC visit**				χ^2^=162.01, p<0.001
<4 months	68,864 (24.89)	53.94	46.06	
≥4 months	91,080 (75.11)	33.69	66.31	
**Wanted last birth**				χ^2^=20.00, p<0.05
Wanted then	134,087 (66.01)	51.26	48.74	
Wanted later	30,712 (27.97)	45.11	54.89	
Wanted no more	10,340 (6.02)	46.67	53.33	
**Decision making capacity**				χ^2^=22.02, p<0.01
No	146,584 (34.59)	53.21	46.79	
Yes	100,136 (65.41)	47.07	52.93	
**Wife beating attitude**				χ^2^=107.32, p<0.001
Agreed for wife beating	120,487 (28.60)	59.26	40.74	
Disagreed for wife beating	126,324 (71.40)	45.04	54.96	
**Barrier to healthcare services**				χ^2^=82.83, p<0.001
No	83,661 (30.50)	40.57	59.43	
Yes	154,366 (69.50)	53.01	46.99	
**Place of residence**				χ^2^=801.94, p<0.001
Urban	83,181 (64.71)	35.82	64.18	
Rural	173,778 (35.29)	72.71	27.29	

### Utilization of deworming medication

**Fig. 1:**
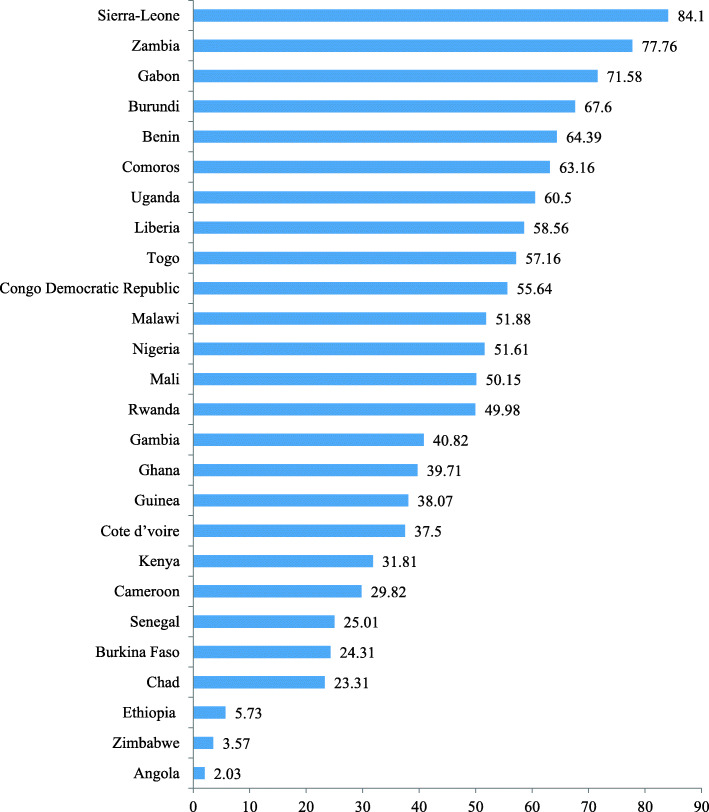
Utilization coverage of deworming medication among married women: evidence from 25 SSA countries DHSs

The pooled results show that more than half (50.74%, 95% CI 48.15–53.31%) of married women in the studied countries in SSA took deworming medications. As shown in Fig. 1, highest utilization coverage of deworming medication were seen in Sierra-Leone (84.1%), Zambia (77.8%), and Gabon (71.6%) respectively, and lowest coverage was reported in Angola (2%), Zimbabwe (3.6%), and Ethiopia (5.7%) respectively (Fig. 1).

As shown in Table 3, wide differences in utilization of deworming medication across sub-African regions are seen with highest coverage in East Africa (67.6%, 95% CI 66.0–69.1%) region followed by Central Africa (50.7%, 95% CI 48.2–53.3%) and lowest coverage in West Africa (24.3%, 95% CI 22.4–26.4%) (Table 3).

### Utilization of deworming medication across explanatory variables

**Fig. 2:**
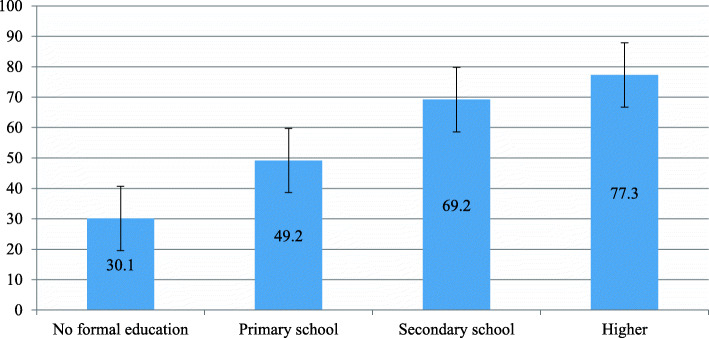
Utilization of deworming medication among pregnant women based on educational level: evidence from 26 sub-Saharan African countries DHSs

Table 2 shows variations in utilization coverage of deworming medication across explanatory variables and subcategories. For instance, utilization of deworming medication varied from 30.1 to 77.3% between married women who had not attended formal education and those who had attended higher education, respectively (Fig. 2).

**Table 3:** Coverage of utilization of deworming medication among pregnant married women by sub-regions: evidence from 26 sub-Saharan African countries DHSs

Sub-regions	Included countries	Pooled sub-regional coverage
**West Africa**	Burkina Faso	24.3% (95% CI 22.4–26.4%)
	Benin	
	Côte d’Ivoire	
	Ghana	
	Gambia	
	Guinea	
	Liberia	
	Mali	
	Nigeria	
	Sierra-Leone	
	Senegal	
	Togo	
**Central Africa**	Angola	50.7% (95% CI 48.2–53.3%)
	Congo Democratic Republic	
	Cameroon	
	Gabon	
	Chad	
**East Africa**	Burundi	67.6% (95% CI 66.0–69.1%)
	Ethiopia	
	Kenya	
	Comoros	
	Malawi	
	Rwanda	
	Uganda	
	Zambia	
	Zimbabwe	

Notes: *CI* confidence interval

**Fig. 3:**
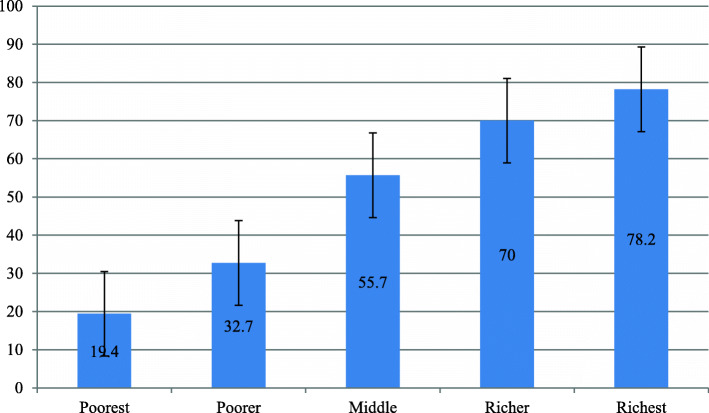
Utilization of deworming medication among pregnant women based on household economic status: evidence from 26 sub-Saharan African countries DHSs

Similarly, among married women between poorest and richest subpopulation, utilization of deworming medication varied from 19.4 to 78.2%, respectively (Fig. 3).

About 61.5% of married women who had media exposure were taking deworming medication, while not more than of 25% of married women who had no media exposure took deworming medication. Moreover, about 61.5% of married who were exposed to media, could it be newspaper, radio, or television, took deworming medication, but the coverage lowered to 25% among married women with no media exposure. Utilization of deworming medication also varied from 46.1 to 66.3% between married women with less than four ANC visits and four and above ANC visits respectively (Table 2).

### Factors associated with utilization of deworming medication

Table 4 shows bivariate and multivariable logistic regression analysis of factors associated with utilization of deworming medication. The bivariate analysis shows that women’s age, women’s educational status, husband’s educational status, women’s occupation, wealth index, media exposure, family size, place of residence, barriers to healthcare services, religion, timing of ANC visit, number of ANC visit, wanted last birth, wife beating attitude, and decision making were associated with utilization of deworming medication. However, the multivariable analysis shows that women’s age, husband’s educational status, wealth index, media exposure, and number of ANC visit were factors associated with utilization of deworming medication.

**Table 4:** Bivariate and multivariable logistic regression output for factors associated with utilization of deworming medication among married women: evidence from 26 SSA countries DHSs

Variables	Model I cOR [95% CI)	P-value	Model II aOR [95% CI)	P-value
**Age in years**				
15–19	Ref		Ref	
20–24	1.55 (1.17–2.05)	**0.002**	1.27 (0.91–1.78)	0.147
25–29	2.09 (1.59–2.74)	**p<0.001**	1.57 (1.13–2.18)	**0.007**
30–34	1.99 (1.48–2.67)	**p<0.001**	1.58 (1.10–2.28)	**0.013**
35–39	1.91 (1.42–2.57)	**p<0.001**	1.93 (1.32–2.84)	**0.001**
40–44	1.84 (1.26–2.70)	**0.002**	1.92 (1.25–2.95)	**0.003**
45–49	0.97 (0.59–1.60)	0.914	1.15 (0.63–2.11)	0.630
**Women’s educational status**				
No formal education	Ref		Ref	
Primary school	2.24 (1.84–2.73)	**p<0.001**	1.03 (0.81–1.30)	0.791
Secondary school	5.21 (4.15–6.54)	**p<0.001**	1.04 (0.77–1.39)	0.786
Higher	7.89 (4.73–13.16)	**p<0.001**	1.00 (0.53–1.88)	0.992
**Husband’s educational status**				
No formal education	Ref		Ref	
Primary school	1.16 (0.91–1.47)	0.214	1.10 (0.86–1.40)	0.435
Secondary school	3.40 (2.76–4.17)	**p<0.001**	1.40 (1.11–1.77)	**0.004**
Higher	6.02 (4.13–8.79)	**p<0.001**	1.28 (0.86–1.91)	0.214
**Women’s occupation**				
Not working	Ref		Ref	
Professional/technical/managerial	1.44 (0.94–2.20)	0.085	0.86 (0.53–1.40)	0.554
Agricultural	0.26 (0.21–0.33)	**p<0.001**	0.85 (0.67–1.07)	0.181
Manual	1.08 (0.72–1.62)	0.695	0.86 (0.54–1.36)	0.521
Others	1.27 (1.03–1.57)	**0.024**	0.99 (0.78–1.25)	0.978
**Wealth index**				
Poorest	Ref		Ref	
Poorer	2.02 (1.54–2.65)	**p<0.001**	1.32 (0.97–1.80)	0.071
Middle	5.22 (3.87–7.04)	**p<0.001**	1.77 (1.24–2.51)	**0.001**
Richer	9.73 (7.11–13.31)	**p<0.001**	2.39 (1.55–3.69)	**p<0.001**
Richest	14.90 (10.40–21.35)	**p<0.001**	3.12 (1.95–4.99)	**p<0.001**
**Media exposure**				
No	Ref		Ref	
Yes	4.77 (3.96–5.76)	**p<0.001**	1.46 (1.18–1.80)	**p<0.001**
**Family size**				
<5	Ref		Ref	
5+	1.28 (1.09–1.49)	**0.002**	1.13 (0.93–1.39)	0.197
**Place of residence**				
Urban	Ref		Ref	
Rural	0.20 (0.16–0.26)	**p<0.001**	0.98 (0.74–1.30)	0.935
**Barriers to healthcare services**				
No	Ref		Ref	
Yes	0.60 (0.50–0.72)	**p<0.001**	0.87 (0.72–1.07)	0.200
**Religion**				
Christian	Ref		Ref	
Muslim	1.08 (0.29–3.98)	0.897	1.73 (0.23–12.94)	0.592
Others	0.51 (0.35–0.74)	**p<0.001**	1.04 (0.71–1.51)	0.824
**ANC follow-up**				
No	Ref		-	-
Yes	39.74 (26.60–59.37)	**p<0.001**	-	-
**Timing of ANC visit**				
>3 months	Ref		Ref	
≤3 months	1.57 (1.32–1.86)	**p<0.001**	1.09 (0.91–1.31)	0.303
**Number of ANC visit**				
<4 month	Ref		Ref	
≥4 month	2.30 (1.92–2.76)	**p<0.001**	1.51 (1.24–1.83)	**p<0.001**
**Wanted last birth**				
Wanted then	Ref		Ref	
Wanted later	1.27 (1.08–1.51)	**0.004**	1.01 (0.83–1.22)	0.901
Wanted no more	1.20 (0.87–1.64)	0.249	1.06 (0.75–1.48)	0.731
**Wife beating attitude**				
Agreed for wife beating	Ref		Ref	
Disagreed for wife beating	1.77 (1.49–2.11)	**p<0.001**	1.19 (0.99–1.43)	0.056
**Decision-making capacity**				
No	Ref		Ref	
Yes	1.27 (1.07–1.52)	**0.007**	1.10 (0.90–1.35)	0.340

Notes: *Ref* reference

Hyphen (-) indicates not included variable at model II due to small proportion of subgroups

More specifically, we found higher odds of utilization of deworming medication among married women within the age groups of 25–29 years (aOR=1.57, 95% CI 1.13–2.18), 30–34 years (aOR=1.58, 95% CI 1.10–2.28), 35–39 years (aOR=1.93, 95% CI 1.32–2.84), and 40–44 years (aOR=1.92, 95% CI 1.25–2.95) compared to married women within 15–19 years of age. The study shows higher odds of utilization of deworming medication among married women whose husband attended secondary school (aOR=1.40, 95% CI 1.11–1.77) compared to married women whose husband did not attend formal education.

Moreover, higher odds of utilization of deworming medication were observed among married women in the middle (aOR=1.77, 95% CI 1.24–2.51), richer (aOR=2.39, 95% CI 1.55–3.69), and richest (3.12, 95% CI 1.95–4.99) households compared to married women in the poorest households. We found higher odds of utilization of deworming medication among married women who had media exposure (aOR=1.46, 95% CI 1.18–1.80) compared to married women who had no media exposure. Finally, in this study, there were higher odds of utilization of deworming medication among married women who had four and above ANC visits (aOR=1.51, 95% CI 1.24–1.83) compared to married women who had less than four ANC visits (Table 4).

## Discussion

In this study, using nationally representative data, we investigated the coverage and sociodemographic, socioeconomic, and women empowerment-related factors associated with deworming medication utilization among pregnant married women in 26 sub-Saharan African countries. The pooled result shows that more than half (50.74%) of married women in the studied countries took deworming medications. The finding from this study was higher compared to the study conducted among 49 STH endemic countries that showed 23% of pregnant women received deworming medication [5]. The variations in the coverage of utilization of deworming medication between these two studies might partly relate to variation in methodology of the studies (married women versus all reproductive age women, and study area/included countries). In this study, utilization coverage of deworming medication varied from as high as 84.1% in Sierra-Leone to as low as 2% in Angola. Concerning sub-African region variations, highest and lowest coverage of utilization of deworming medication among pregnant married women was seen in East Africa (67.6%) and West Africa (24.3%) regions respectively. The variations across African regions and countries might be related to factors associated with utilization of deworming medication such as socioeconomic factors [16–18] and time effect (the year when the DHS data was collected across countries; 2010 to 2019).

Women’s age was associated with utilization of deworming medication. More specifically, we found higher odds of utilization of deworming medication among older married women compared to adolescent married women. The plausible reason could be higher educational and career accomplishment as well as better family income which increase with age [37, 38]. Older women usually are proactive, and they emphasize on prevention of diseases [37, 39], which could be related to their previous experience [37].

The study showed higher odds of utilization of deworming medication among married women whose husbands were educated, compared to married women whose husbands did not have formal education. Women with educated husbands have better uptake of maternal health services due to the fact that educated husbands participate in promoting their wives’ health and have positive relationship with their wives [40]. In addition, educated husbands are more likely to encourage their wives to seek and utilize healthcare services during pregnancy [41]. On the other hand, husbands with no formal education may prevent their wives from utilizing health services due to being uncomfortable with touching or examining their wives [42]. The other reason about husband’s education effect is its linkage with income and wealth [43]. Scholars have documented that education is the noticeable factor to get employment opportunities, earn better, and increase individual, communities, and national economic growth [44–46] that again has positive influence in accessing healthcare services [47, 48]. Hence, to use the positive impact of husband education, increasing national education coverage, husband-oriented health education programs, and involvement of husband in maternal healthcare services may be the policy recommendations [49, 50].

Moreover, the study showed higher odds of utilization of deworming medication among married women from higher socio-economic households compared to married women in the poorest households. This finding is comparable with a prior study in Ghana [51]. Compared to wealthier women, economically disadvantaged women encounter problems in accessing healthcare services due to direct costs such as payment for medication that may include the possibility of paying for medication in the time when stock runs out from the dispensary and indirect costs such as transport costs and unpaid working hours [40, 47, 48, 52]*.* Unfortunately, the burden of helminthic infection and its consequences, anemia, is more prevalent among individual or communities within countries of low socioeconomic status such as South East Asia and Africa [53–55]. Therefore, giving attention to women in economically disadvantaged households could be one intervention mechanism for enhancement of uptake of deworming medication.

Pregnant women need correct information about healthcare services, and it can help them to have information about the available healthcare services and their needs through visual appealing, audio message, and printed reports [42, 56]. In this study, we found higher odds of utilization of deworming medication among married women who were exposed to media, compared to married women who had no media exposure. Prior studies in India [57], Bangladesh [58], South Asia [42], Uganda [59], and Malawi [60] documented the influence of media on maternal health services. Mass media could have tremendous role in the dissemination of health-related information and development of knowledge for mothers especially for those population groups with less education and those who live in rural setting [42, 57]. Therefore, media-focused regional and national public health policies and practices including prepare campaign and ongoing health education programs using mass media need to be considered to increase the uptake of deworming medication [61].

We found higher odds of utilization of deworming medication among married women who had four and above ANC visits compared to married women who had less than four ANC visits. ANC visit is a good opportunity to receive deworming medication because more information about benefits of deworming and positive pregnancy outcomes are gained through counseling during repeated ANC visits [13]. Therefore, governments should reduce or eliminate barriers that limit number of ANC visits such as long distance to health facilities that may be aggravated by difficult geographical/road conditions [62], and strengthening the use of ANC services in the region as newly recommended by WHO is expected from the health sector and needs to become an agent to coordinate and work with other sectors so as to enhance uptake of deworming medication among pregnant women.

In this study, women’s decision-making power and wife beating attitude was not associated with uptake of deworming medication. This might indirectly tell us access to and utilization of maternal healthcare services among married women are strongly determined by husband educational level [63]. Moreover, gender inequity such as husband’s financial status within the household might determine the uptake of maternal health services [64]. In fact, getting permission from husband is one of the four main barriers to access healthcare services [47, 48]. Several prior studies also documented the strong association between husband education and/or involvement with uptake of maternal health service utilization [65–67]. Therefore, considering designing policies that target on enhancing husband education coverage and involvements help to increase the uptake of maternal health services including deworming medication [64, 67].

### Strengths and limitations of the study

Determining utilization coverage of deworming medication and investigating its demographic, socioeconomic, and women empowerment-related factors among pregnant married women using nationally representative, large sample size and across several countries (25 countries in SSA) is the main strength of this study. The findings of this study could be used by policy makers to improve the uptake of deworming medication in SSA. In terms of limitations, although we attempted to incorporate many countries, due to the inclusion criteria, we only included few countries in SSA, and the findings may not be generalizable to all sub-Saharan African countries. Moreover, since we were interested in examining women empowerment-related factors, which are applicable to married women only, we could not include non-married women, and therefore, findings are limited to married women. Third, the cross-sectional nature of the study could not allow for inferring causal-effect relationship. Lastly, the time when the surveys were conducted varied by up to 9 years across studied countries and that needs to be considered as it may affect the comparisons due to the time effect.

## Conclusion

The pooled results show that about half of married women received deworming medication during their pregnancy. Women’s age, husband’s educational status, wealth index, media exposure, and number of ANC visits were factors associated with utilization of deworming medication. Therefore, enhancing women’s education, involving men in women’s health programs, accessing women’s use of media especially those who are not literate and living in rural areas and disseminating information about maternal healthcare services through mass media, and ensuring that economically disadvantaged households benefit from national economic growth can be considered as policy measures. Moreover, providing more attention to adolescent or young pregnant women and strengthening ANC services including increasing number of ANC visits can be another solution for increased uptake of deworming medication among married pregnant women especially in West African countries.

## Data Availability

Data for this study were sourced from the Demographic and Health surveys (DHS) and available here: http://dhsprogram.com/data/available-datasets.cfm.

## Abbreviations

CI: Confidence intervals
DHS: Demographic and Health Surveys
STH: Soil-transmitted helminthiasis
WHO: World Health Organization
